# Developing optimal search strategies for detecting clinically sound and relevant causation studies in EMBASE

**DOI:** 10.1186/1472-6947-5-8

**Published:** 2005-03-22

**Authors:** R Brian Haynes, Monika Kastner, Nancy L Wilczynski, the Hedges Team

**Affiliations:** 1 Health Information Research Unit, Department of Clinical Epidemiology and Biostatistics, McMaster University, Hamilton, Ontario, L8N 3Z5, Canada; 2 Department of Medicine, Faculty of Health Sciences, McMaster University, Hamilton, Ontario, L8N 3Z5, Canada

## Abstract

**Background:**

Evaluating the existence and strength of an association between a putative cause and adverse clinical outcome is complex and best done by assessing all available evidence. With the increasing burden of chronic disease, greater time demands on health professionals, and the explosion of information, effective retrieval of best evidence has become both more important and more difficult. Optimal search retrieval can be hampered by a number of obstacles, especially poor search strategies, but using empirically tested methodological search filters can enhance the accuracy of searches for sound evidence concerning etiology. Although such filters have previously been developed for studies of relevance to causation in MEDLINE, no empirically tested search strategy exists for EMBASE.

**Methods:**

An analytic survey was conducted, comparing hand searches of journals with retrievals from EMBASE for candidate search terms and combinations. 6 research assistants read all issues of 55 journals indexed in EMBASE. All articles were rated using purpose and quality indicators and categorized into clinically relevant original studies, review articles, general papers, or case reports. The original and review articles were then categorized as 'pass' or 'fail' for scientific merit according to explicit criteria in the areas of causation (etiology) and other clinical topics. Candidate search strategies were developed for causation, then run in a subset of 55 EMBASE journals, the retrievals being compared with the hand search data. The sensitivity, specificity, precision, and accuracy of the search strategies were calculated.

**Results:**

Of the 1489 studies classified as causation, 14% were methodologically sound. When search terms were combined, sensitivity reached 92%. Compared with the best single-term strategy, the best combination of terms resulted in an absolute increase in sensitivity (19%) and specificity (5.2%). Maximizing specificity for combined terms resulted in an increase of 7.1% compared with the single term but this came at an expense of sensitivity (39% absolute decrease). A search strategy that optimized the trade-off between sensitivity and specificity achieved 81.9% for sensitivity and 81.4% for specificity.

**Conclusion:**

We have discovered search strategies that retrieve high quality studies of causation from EMBASE with high sensitivity, high specificity, or an optimal balance of each.

## Background

Clinical problems encountered by clinicians often involve examining questions about harm that involve genes, treatments, or environmental exposures [1,2]. Knowledge of a causal relationship is important to clinicians, as it guides their approach to better patient management, and provides recommendations for future research on modifiable environmental risk factors or genetically determined characteristics [3]. With the increasing burden of chronic disease and greater time demands on clinicians and the explosion of research information, effective retrieval of the best evidence has become difficult. Clinicians seldom know of the relevant and rigorous evidence that is available on a particular topic and most often do not attempt to retrieve it even when pertinent to a clinical problem at hand [4,5].

Large biomedical databases such as MEDLINE and EMBASE provide online access to the medical literature [6], and conducting searches in these databases has been recommended as a basic skill for evidence-based practitioners [7]. To make better clinical decisions with the potential of positively affecting the care of their patients, clinicians need ways to optimize their retrieval of the best evidence [8-10]. However, clinicians face a number of obstacles that inhibit optimum search retrieval. The overwhelming amount of available information, coupled with the over 2 million new articles that get published each year [10-12], makes keeping up-to-date challenging and difficult [10,13]. In EMBASE, the European biomedical database counterpart to MEDLINE, clinicians must search through more than 9 million citations from over 4000 journals to narrow their search for best evidence [14].

For clinicians, increasing time demands restrict the practice of evidence-based medicine [4,7], despite the strong belief in its implementation [6,15]. Lack of time is also a major barrier to conducting searches [4,9,15]. Even though the evidence is readily available, clinicians are more likely to seek answers from colleagues [5] or other easily accessible resources than to search for answers with evidence and evaluate the results of original research [16]. As a result, most clinicians do not find answers to their clinical questions or do not pursue them because they have doubt about the existence of useful information in available resources [4,9].

The very low concentration of rigorous studies also limits clinicians' awareness and detection of key articles [13]. Furthermore, clinicians use less than optimal strategies because they lack search skills; do not know how to narrow their searches without missing relevant information; and have uncertainties about when to stop searching, which articles to read, and how thoroughly to read them [16,17].

Methodologic search filters (which capture relevant articles while eliminating those that are not of interest) are one way of improving the retrieval of scientifically sound and clinically relevant studies from biomedical literature databases [18]. Search strategies are useful tools and have been developed for causation studies as well as for studies in other categories (e.g., treatment) for MEDLINE [19,20]. For EMBASE however, very few search strategies have been developed [21]. In fact, we are unable to find an empirically tested search strategy for the retrieval of causation studies in EMBASE.

In this paper, we report on the evaluation and comparison of the retrieval performance of causation search strategies in EMBASE with a manual review ("gold standard") of each article for each issue of 55 journals in 2000. Compared with previous strategies developed for MEDLINE in 1991, the methods we applied for selecting articles for EMBASE were tighter and the calibration database larger (55 journals for EMBASE compared with 10 for MEDLINE in 1991). In addition, we tested many more search strategies, which for MEDLINE resulted in the development of search strategies that work better than the ones previously reported. The focus of the strategies is to help clinicians and researchers retrieve methodologically sound study reports on causation, to assist with evidence-based patient care decisions based on the best quality evidence available. To our knowledge, no approach exists that applies such rigorous standards to EMBASE.

## Methods

The study compared the retrieval performance of methodologic search terms and phrases in EMBASE with a manual review of each article for each issue of 55 journal titles for the year 2000. Index terms and text words related to research design features were run as search strategies. The search strategies were treated as "diagnostic tests" for sound studies and the manual review of the literature was treated as the "gold standard." The sensitivity, specificity, precision, and accuracy of EMBASE search strategies were determined. Sensitivity for a given topic is defined as the proportion of high quality articles for that topic that are retrieved; specificity is the proportion of low quality articles not retrieved; precision is the proportion of retrieved articles that are of high quality; and accuracy is the proportion of all articles that are correctly classified.

Individual search terms with sensitivity > 25% and specificity > 75% for causation studies were incorporated into the development of search strategies that included a combination of 2 or more terms. All combinations of terms used the Boolean OR, for example, "risk.tw. OR cohort.tw.". The Boolean AND was not used because this strategy invariably compromised sensitivity. For the development of multiple-term search strategies to either optimize sensitivity or specificity, we tested all 2-term search strategies with sensitivity at least 75% and specificity at least 50%. For optimizing accuracy, 2-term search strategies with accuracy > 75% were considered for multiple-term development. 13,901 search strategies were tested.

We did not attempt to use logistic regression to improve search performance in this study because our previous development of regression strategies for retrieving studies of treatment [unpublished observation] and prognosis [22] showed no benefit.

Six research assistants hand searched 170 journals titles in total for the year 2000, and applied methodologic criteria to each item in each issue to determine if the article was methodologically sound for 7 purpose categories (e.g., causation, treatment, diagnosis; two other types of articles, cost and qualitative studies, were also classified but had no rigor criteria). All purpose category definitions and corresponding methodologic rigor were outlined in a previous paper [23]. The methodologic criteria applied for studies of causation are in Table 1. Research staff were rigorously calibrated before reviewing the 2000 literature and inter-rater agreement for application of all criteria exceeded 80% beyond chance [23].

**Table 1:** Methodologic Rigor Applied for Studies of Causation

Purpose Category	Methodologic Rigor
Causation	Observation concerned with the relationship between exposures and putative clinical outcomes; Data collection is prospective; Clearly identified comparison group(s); Blinding of observers of outcome to exposure; and Analysis consistent with study design.

The 170 journal titles reviewed were chosen based on recommendations of clinicians and librarians, Science Citation Index Impact Factors provided by the Institute for Scientific Information, and ongoing assessment of their yield of studies and reviews of scientific merit and clinical relevance for the disciplines of internal medicine, general medical practice, mental health, and general nursing practice (list of journals provided by the authors upon request). 135 of the 170 journals were indexed in EMBASE. We previously developed search strategies in MEDLINE using the 161 hand-searched journals that were indexed in MEDLINE but found that search strategies developed in much smaller journal subsets are equally robust [24] and that computation time is substantially decreased. We also found that when strategies were developed in 60% of the database and validated in the remaining 40% there were no statistical differences in performance [19]. Thus, for EMBASE we developed search strategies using a 55 journal-subset chosen based on those journals, which had the highest number of methodologically sound studies. This selection somewhat enriches the sample of target articles (those that "pass" for scientific merit) thereby improving the estimates of the sensitivity and specificity search term performance and simplifying data processing. Enriching the prevalence of qualified articles, however, results in overestimates of precision and, to a lesser extent, accuracy. This problem is universal in using a diagnostic testing approach, and is also true for any other classification approach of which we are aware, including machine learning models.

To identify candidate search terms and strategies, we compiled an initial list of index terms and text words by selecting words that related to etiology (eg, etiology, cause, causation) and to research methods for establishing causation (see examples below). We then sought input from clinicians and librarians in the United States and Canada through interviews of known searchers, and requests at meetings and conferences. Individuals were asked to identify terms or phrases they used when searching for studies of causation, prognosis, diagnosis, treatment, economics, clinical prediction guides, reviews, costs, and studies of a qualitative nature. We compiled a list of 5385 terms of which 4843 were unique and 3524 returned results in the 55-journal subset in EMBASE (list of terms tested provided by the authors upon request). Examples of the search terms tested are 'adverse drug reaction', 'risk ratio', 'cohort study', and 'harm', all as text words; 'risk', the index term, and the index term 'exposure', exploded.

## Results

Indexing information was downloaded from EMBASE for 27,769 articles from the 55 hand searched journals. Of these, 1489 were classified as causation, of which 215 (14.4%) were judged methodologically sound. Search strategies were developed using all 27,769 articles. Thus, the strategies were tested for their ability to retrieve articles about higher quality causation studies from all other articles, including both lower quality causation studies and all non-causation studies.

The operating characteristics of the best single-term for high-sensitivity, high-specificity, and best optimization of sensitivity and specificity are displayed in Table 2. When specificity was maximized (87.5%), the most noticeable, but expected trade-off was the decrease in sensitivity (21.9% absolute decrease), but there was a slight increase in precision (1.8% absolute increase).

**Table 2:** Single Term with the Best Sensitivity (keeping Specificity ≥50%), Best Specificity (keeping Sensitivity ≥50%), and Best Optimization of Sensitivity and Specificity (based on the lowest possible absolute difference between sensitivity and specificity) for Detecting Studies of Causation in EMBASE in 2000

Search term OVID search*	**Sensitivity (%) (n = 215)**	**Specificity (%) (n = 27,554)**	**Precision (%)†**	**Accuracy (%) (n = 27,769)**
**Best sensitivity ** exp general aspects of disease	72.6 (66.6 to 78.5)	55.7 (55.1 to 56.3)	1.3 (1.1 to 1.5)	55.8 (55.2 to 56.4)
**Best specificity ** exp risk	50.7 (44.0 to 57.4)	87.5 (87.1 to 87.8)	3.1 (2.5 to 3.6)	87.2 (86.8 to 87.6)
**Best Optimization of Sensitivity & Specificity ** control:.mp.	60.9 (54.5 to 67.5)	66.4 (65.9 to 67.0)	1.4 (1.2 to 1.6)	66.4 (65.8 to 66.9)

*The search strategy is reported using Ovid's search engine syntax for EMBASE.

†Denominator varies by row.

exp = explode, a search term that automatically includes closely related indexing terms; : = truncation; mp = multiple posting – term appears in title, abstract, or subject heading.

Combinations of terms with the best results for sensitivity, specificity, and optimization of sensitivity and specificity are shown in Table 3. As expected, combining terms increased sensitivity. The 3-term combination strategy, "risk:.mp OR exp methodology OR exp epidemiology" yielded the best sensitivity (91.6%) with specificity 60.9%. Compared with the best sensitivity single-term strategy, "exp general aspects of disease", the combination strategy resulted in an absolute increase in both sensitivity (19%) and specificity (5.2%).

**Table 3:** Combination of Terms with the Best Sensitivity (keeping Specificity ≥50%), Best Specificity (keeping Sensitivity ≥50%), and Best Optimization of Sensitivity and Specificity (based on abs [sensitivity-specificity]<1%) for Detecting Studies of Causation in EMBASE in 2000

Search Strategy OVID search*	**Sensitivity (%) (n = 215)**	**Specificity (%) (n = 27,554)**	**Precision (%)‡**	**Accuracy (%) (n = 27,769)**
**Best Sensitivity** risk:.mp. OR exp methodology OR exp epidemiology	91.6 (87.9 to 95.3)	60.9 (60.3 to 61.4)	1.8 (1.6 to 2.0)	61.1 (60.5 to 61.7)
**Best Specificity** cohort.tw. OR relative risk:.tw.	53.0 (46.4 to 59.7)	94.6 (94.4 to 94.9)	7.1 (5.9 to 8.4)	94.3 (94.0 to 94.6)
**Small decrease in best specificity with a substantive increase in sensitivity** cohort.tw. OR relative risk:.tw. OR adjusted OR†.tw.	61.4 (54.9 to 67.9)	92.9 (92.6 to 93.2)	6.3 (5.3 to 7.3)	92.6 (92.3 to 92.9)
**Best Optimization of Sensitivity & Specificity** risk.tw. OR mortalit:.tw. OR cohort.tw.	81.9 (76.7 to 87.0)	81.4 (80.9 to 81.8)	3.3 (2.8 to 3.8)	81.4 (80.9 to 81.8)

*Search strategies are reported using Ovid's search engine syntax for EMBASE.

†OR = odds ratio.

‡Denominator varies by row.

: = truncation; mp = multiple posting – term appears in title, abstract, or subject heading; exp = explode, a search term that automatically includes closely related indexing terms; tw = textword (word or phrase appears in title or abstract).

The two-term strategy, "cohort.tw. OR relative risk:.tw." yielded the best specificity (94.6%) but with an expected trade-off in sensitivity, which was lowered to 53% (38.6% absolute decrease). However, maximizing specificity improved both precision (5.3% absolute increase) and accuracy (33.2% absolute increase). The combination of 3 terms, "cohort.tw. OR relative risk:.tw. OR adjusted OR.tw" (where "adjusted OR" is not the Boolean OR but rather refers to adjusted odds ratio) achieved a substantive increase in sensitivity (8.4% absolute increase) with a small decrease in specificity (1.7% absolute decrease) (Table 3). The combination of search terms, "risk.tw. OR mortalit:.tw. OR cohort:.tw." (81.9% sensitivity, 81.4% specificity) led to the best optimization of sensitivity and specificity (Table 3).

Table 4 shows the 3 top-performing search strategies for best sensitivity, best specificity, and best balance between sensitivity and specificity. Because the accuracy of search terms is driven by their specificity, the 3 top-performing search strategies with the best accuracy were similar to those with best specificity. In addition, two 2-term strategies slightly outperformed all the 3-term strategies for best specificity.

**Table 4:** Top 3-performing Combination of Terms with the Best Sensitivity (keeping Specificity ≥50%), Specificity (keeping Sensitivity ≥50%), and Best Optimization of Sensitivity and Specificity (based on abs [sensitivity-specificity]<1%) for Detecting Studies of Causation in EMBASE in 2000

Search Strategy OVID search*	**Sensitivity (%) ** **(n = 215)**	**Specificity (%)** ** (n = 27,554)**	**Precision (%)†**	**Accuracy (%)** ** (n = 27,769)**
**Top 3-performing combination of terms with best Sensitivity**				

risk:.mp. OR exp methodology OR exp epidemiology	91.6 (87.9 to 95.3)	60.9 (60.3 to 61.4)	1.8 (1.6 to 2.0)	61.1 (60.5 to 61.7)
risk:.tw. OR exp methodology OR exp epidemiology	91.2 (87.4 to 95.0)	63.0 (62.4 to 63.6)	1.9 (1.6 to 2.2)	63.2 (62.6 to 63.8)
risk:.mp. OR exp methodology OR exp mortality	90.7 (86.8 to 94.6)	65.1 (64.5 to 65.7)	2.0 (1.7 to 2.3)	65.3 (64.7 to 65.8)

**Top 3-performing combination of terms with best Specificity**				

cohort.tw. OR relative risk:.tw.	53.0 (46.4 to 59.7)	94.6 (94.4 to 94.9)	7.1 (5.9 to 8.4)	94.3 (94.0 to 94.6)
confidence interval.tw. OR relative risk:.tw.	50.7 (44.0 to 57.4)	94.5 (94.2 to 94.7)	6.7 (5.4 to 7.9)	94.1 (93.8 to 94.4)
OR relative risk:.tw. OR cohort:.tw.	53.5 (46.8 to 60.2)	94.4 (94.1 to 94.6)	6.9 (5.7 to 8.1)	94.1 (93.8 to 94.3)

**Top 3-performing combination of terms with best optimization of Sensitivity and Specificity**				

risk.tw. OR mortalit:.tw, OR cohort.tw.	81.9 (76.7 to 87.0)	81.4 (80.9 to 81.8)	3.3 (2.8 to 3.8)	81.4 (80.9 to 81.8)
risk:.tw. OR cohort:.mp. OR confidence interval:.tw.	81.9 (76.7 to 87.0)	81.2 (80.8 to 81.7)	3.3 (2.8 to 3.8)	81.3 (80.8 to 81.7)
risk.tw. OR mortalit:.tw. OR cohort:.tw	81.9 (76.7 to 87.0)	81.2 (80.8 to 81.7)	3.3 (2.8 to 3.8)	81.2 (80.8 to 81.7)

*Search strategies are reported using Ovid's search engine syntax for EMBASE.

†Denominator varies by row.

: = truncation; mp = multiple posting – term appears in title, abstract, or subject heading; exp = explode, a search term that automatically includes closely related indexing terms; tw = textword (word or phrase appears in title or abstract).

## Discussion

We developed causation search filters for EMBASE that provide for highly sensitive, highly specific, and highly accurate searches in EMBASE for high quality studies concerning etiology. The utility of these 3 types of search filters will vary according to the needs of end users or the clinical question that is being sought.

For example, a person conducting a search to find original articles for constructing a systematic review will have different retrieval needs than the clinician who is looking for quick answers to manage a patient. The best sensitive search would be more beneficial for a systematic review. Although it is time consuming to search through 270 citations that may include some irrelevant articles, key studies that are needed to conduct a robust systematic review would not be missed. In contrast, for quick answers, the narrower yield of a specific search takes less time, and will likely provide a sufficient number of relevant articles to answer the clinical question sought, but with somewhat higher potential for missing key studies. The trade-off between time investment and consequences of missing useful evidence is important to consider [21].

Our results indicate that combination-term strategies generally perform better than single-term strategies. However, in our previous research, "risk:.mp", yielded close to best sensitivity in developing causation search filters in MEDLINE [19]. The resulting test characteristics were surprising, as this search resulted in a substantial gain in specificity (26.5% absolute increase) at a very low cost to sensitivity (0.5% absolute decrease). An end user who doesn't have adequate time for a lengthy search will sacrifice only a small decrease in best sensitivity in exchange for a much higher specificity. To test if a similar benefit could be achieved in sensitivity, we also tested the best specificity single-term strategy from our previous MEDLINE strategy, "Risk factor:.mp" in EMBASE. Unfortunately, the small gain in specificity (5.3% absolute increase) was at a very high cost to sensitivity, which was lowered to 35.8% – well below our acceptable prespecified sensitivity at ≥ 50%. Unfortunately, we were only able to do limited comparisons between EMBASE and MEDLINE search strategies, as the two databases do not support the same index terms.

A logistic regression approach to developing search strategies was done when deriving search filters for MEDLINE [22]. The analysis did not improve on search strategies developed using the Boolean approach described above.

Another expected result from our study was that precision was generally low. For a large, multipurpose biomedical database such as EMBASE, it was not surprising to find a low proportion of relevant, high quality causation studies. Although a slight improvement in precision was seen when specificity was maximized, the overall low precision in our study will still require physicians to invest time eliminating irrelevant articles. However, improving precision may be possible by combining search strategies with content-specific terms using the Boolean "AND" or "AND NOT". Our future research will focus on enhancing precision by developing more sophisticated search filters, and by using the strategies above.

## Conclusion

We developed several search strategies that can enhance the retrieval of causation articles in EMBASE. The needs of end users play an important role in determining the most beneficial trade-off between sensitivity and specificity.

## Competing interests

The authors declare that they have no competing interests.

## Pre-publication history

The pre-publication history for this paper can be accessed here:


